# Endothelial Progenitor Cells and Cardiovascular Correlates

**DOI:** 10.7759/cureus.3342

**Published:** 2018-09-21

**Authors:** Tatenda A Mudyanadzo

**Affiliations:** 1 Surgery, University of South Alabama, Mobile, USA

**Keywords:** endothelial progenitor cells, hypertension and late outgrowth endothelial progenitor cells, angiogenesis, late outgrowth endothelial progenitor cells, diabetes mellitus and late outgrowth endothelial progenitor cells, statin and late outgrowth endothelial progenitor cells

## Abstract

Cardiovascular disease is cited as the underlying cause of death in one out of every three deaths within the United States; this burden on the health care system percolates down to affect patients on an individual level. In part, the problem arises from the low regenerative capacity of cardiovascular system cells, for example, cardiac myocytes, and from oxidative stressors to the human body.

Endothelial progenitor cells (EPCs) are a type of stem cell, and various clinical conditions including hypertension and renal failure underlie their dysfunction. EPCs are classified as either early or late outgrowth endothelial progenitor cells depending on the time they appear in circulation and at the site of injury after an inciting event. Their function is paracrine through the release of cytokines, growth factors and chemokines such as interleukin-6 and vascular endothelial growth factor, and they are involved in transdifferentiation into vascular smooth muscle cells and potentially cardiac myocytes. They are beneficial to the modification of cardiovascular cell apoptosis, fibrosis, and contractility. In times of stress, the normal function of endothelial progenitor cells is altered; this creates a maladaptive cycle where stress and failed coping mechanisms enhance each other toward the culmination of cardiovascular disease.

The development of the cardiovascular system follows gastrulation in the embryonic period, and the cells that form the system are derived from the mesoderm; being mesoderm, the vascular cells exhibit heterogeneity in their origin and function. The need to understand the molecular and cellular regulatory pathways during development can amalgamate efforts of endothelial cell and cardiovascular system pathophysiology for the advancement of patient cardiovascular reserve and function.

## Introduction and background

The development of the vascular system is an early process during embryo- and organogenesis; the heart is the first organ that becomes functional in the human embryo. Development of the heart begins in the cardiac bulge of the embryo; this along with the vascular system develop from the mesoderm during the gastrulation stage of embryogenesis. Vasculogenesis, the development of the vascular system within the mesoderm, progresses through the formation of vascular islands that enlarge and coalesce to form the vascular system and hematopoietic cell lines [1]. Marked remodeling of the cardiovascular system (CVS) occurs as the embryo develops through the stages of organogenesis and vasculogenesis; the blood vessels branch, degenerate, and canalize among other remodeling processes that underlie angiogenesis. Angiogenesis involves the differentiation of various parts of the vasculature, including capillaries, veins, arteries, and lymphatic vessels. The development of the cardiovascular system is both intra-embryonic and extra-embryonic (yolk sac and placenta) with development starting primarily in the yolk sac followed by migration of hematopoietic cells to the liver and finally ending up in the bone marrow.

Mesodermal cells, responsible for differentiating into various components of the CVS, arise from the epiblast and give rise to progenitor cells. The progenitor cells differentiate to form, for example, hematopoietic, arterial, capillary and lymphatic components. These cells do not vanish but persist through human life as we know it. These multipotent cells localize and reside within different areas of the CVS such as the bone marrow, media and adventitia layers of the blood vessels [2]; their function during the embryonic period is to generate, differentiate, and perpetuate the developing CVS of the embryo.

During the embryonic period, the endocardium and endothelium progenitor cells bud off and become involved in hematopoiesis [3,4]. Not all cells in these areas are capable of this regenerative capability, and this heterogeneity in the potential of the cells extends beyond embryonic and fetal life to persist into adulthood; this points to the presence of stem cells within the cardiac myocyte pool. Although present, these cardiac myocyte stem cells have low regenerative capacity in adults. Genetic differentiation of cells seems of more importance than local stimuli in the differentiation of cells into those with the capability to regenerate and terminal cells without any regenerative capacity [1]. Some of the multipotent cells transform into smooth muscle cells responsible for the formation of the media and adventitia layers of the vascular system. These migrating and differentiating embryonic smooth muscle cells lay matrix and expand the muscular layers of the developing blood vessels [1]; platelet-derived growth factor and transforming growth factor-beta are some of the chemo-attractants responsible for this migration and proliferation.

In adults, smooth muscle cells retain their potential to migrate and proliferate especially in response to mitogens, stress, and inflammation [1]. This plasticity heralds a transformation from their primary contractile function role into a mesenchymal form where they lay more matrix in the intima and media and proliferate to expand the muscular layer. Although stimulated by pathophysiologic changes, genetic factors underlie this alteration in pathophysiologic function. Studies have reported these alterations in the function of smooth muscle cells that occur when cells are incubated in culture; these changes are noticed, for example, during atherosclerosis and in arteriosclerosis after vein graft [5].

There is growing evidence that other cell types are involved in neovascularization. Cells derived from the bone marrow and other circulating blood cells (such as mononuclear cells) [6] can transform into cells that participate in neovascularization and vascular remodeling [7]; these are endothelial progenitor cells (EPCs) [6].

EPCs can be found circulating in the blood or confined to bone marrow or linings of vessel walls such as the adventitia and media. Historically, the function of these EPCs has been understood to restore the integrity of the endothelium after an insult. However, recent studies indicate a function more varied than this picture; EPCs have been found to be involved in the production of vasodilating agents including nitric oxide [6] and to possess vascular angiogenic properties. This difference between the historical and contemporary views related to the classification of EPCs has significance associated with the time to onset of action from the insulting event [8].

EPCs are currently classified based on culture properties as either early-onset endothelial progenitor cells (EOEPCs) or late-onset endothelial progenitor cells (LOEPCs). EOEPCs have been found elevated in patients one to ten days after an insult such as ischemic stroke and myocardial infarction. Their purpose is the restoration of endothelial function through paracrine means, as they lack direct vasculogenic effects [6,8]. EOEPCs have a hematopoietic phenotype and are not of endothelial lineage [9]. In contrast, LOEPCs peak in numbers approximately four to six weeks post-insult and possess direct vasculogenic effects along with their production of angiogenic factors [9,10]; they share genetic and phenotypic similarities with endothelial cells [9]. Furthermore, EPCs are different in appearance; EOEPCs have a spindle shape whereas LOEPCs have a cobblestone appearance. Flow cytometry and culture techniques can be utilized to identify these different mononuclear cell derivates [6]; however, in practice, the separation and definition of these cell lines have not yet been integrated [9].

## Review

Cardiovascular disease puts a huge economic burden on society; as life expectancy rises, increased prevalence and complexity of these conditions are anticipated. Cardiovascular disease has been indexed as an underlying cause of death in approximately one out of every three deaths in the United States, which translates to roughly one death every 38 seconds attributable to cardiovascular disease [11]. The etiology and progression of these diseases are multifactorial and range from the essentials of our existence, genetics, to external factors such as lifestyle. One molecular problem associated with cardiovascular disease has to do with how the body can regenerate its vasculature and the factors that influence this potential. The purpose of this review is to discuss EPCs and their role in cardiovascular remodeling after endothelial damage; bone marrow-derived cells found in open circulation and involved in the repair and maintenance of vascular endothelium [12-14]. These cells are divided into two groups in this discussion (though some researchers have divided these into more than two groups), EOEPCs and LOEPCs, a differentiation that depends on the time of their appearance in circulation, which in turn determines their function. EOEPCs are hematopoietic and operate via paracrine effects including the release of growth factors, cytokines, and chemokines; whereas LOEPCs are endothelial in phenotype and can differentiate into endothelial cells to produce an autocrine effect that reduces abnormal remodeling and induces apoptosis while enhancing contractility of smooth muscle cells.

Methods

**Figure 1:**
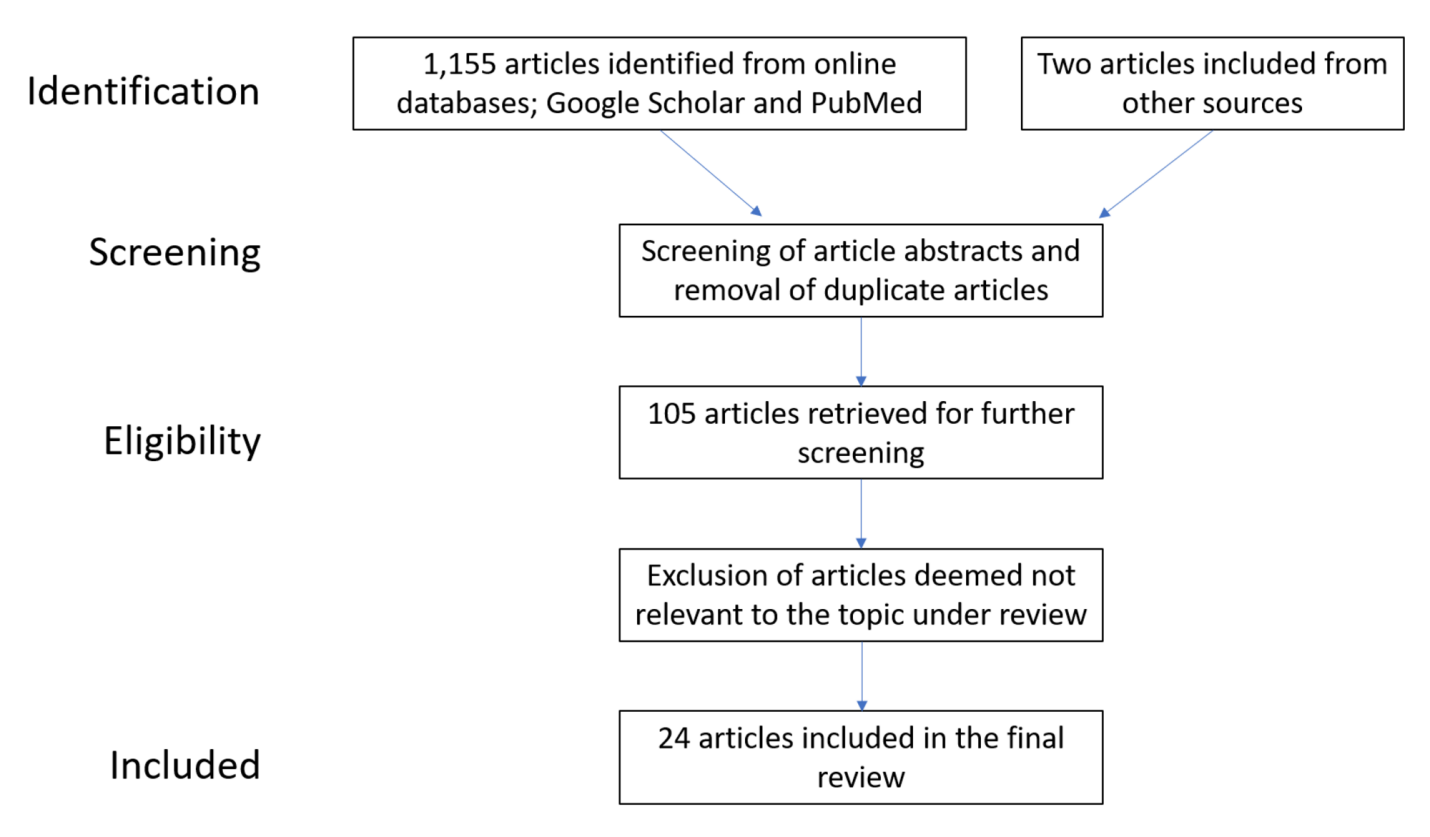
Data collection flow process

The effects of endothelial progenitor cells affect all systems and are an evolving area of research. The method used to obtain the information reported herein was via the use of the free online search engines Google Scholar and PubMed. To identify relevant material, the following keywords were utilized: late outgrowth endothelial progenitor cells, angiogenesis and late outgrowth endothelial progenitor cells, hypertension and late outgrowth progenitor cells, statin and late outgrowth progenitor cells, diabetes and endothelial progenitor cells. Use of these keywords relates to the central issues of this systematic review article: endothelial progenitor cells and cardiovascular correlates. There were no filters to this search as there are not many articles related to the topic under consideration. Screening of the identified articles was performed; articles with the full article available were chosen, while articles not relevant to the topic under consideration or tangential in detail were excluded. Eligible articles were selected to synthesize this qualitative review article. The data extraction process followed the depiction in Figure 1.

Limitations and ethical considerations

The lack of filters can be viewed as a limitation to this research; however, the explanations afforded by these articles and the synthesis of this article are based on the sound foundations of normal disease pathophysiology. Most of the research articles identified were early phase research articles that had a relatively small set of patient numbers. Several studies utilized animal-derived data extrapolated to human studies.

There were no ethical issues to be considered while compiling this review article.

Clinical correlations

On review of the supportive role of EPCs, some studies indicated they are beneficial while some studies indicated the opposite; higher levels of EPCs have been postulated to be beneficial to patients at risk of cardiovascular diseases such as patients with class 2 obesity or higher [6]. This protective role has been researched, and one trial as far as we are aware revealed an increase in EPCs after bariatric surgery at 24 months post-surgery; this contributed to a reduction of cardiovascular risks in this cohort of patients [6]. Experimental results on ischemic muscle have revealed an increase in the blood flow and formation of collateral circulation after administration of LOEPCs. This effect emanates from the angiogenic effects of LOEPCs and to the paracrine effect of their released substrates such as vascular endothelial growth factor (VEGF) [9].

On the other hand, EPCs respond to hypertension, hypercholesterolemia, and a pro-inflammatory environment to cause thickening and loss of compliance in coronary and cerebral blood vessels. These changes are part of the mechanism leading to increased risk of harmful events such as ischemic strokes, myocardial ischemia, and rejection of grafts [8]. The loss of compliance and maladaptive remodeling of vascular walls in response to an inflammatory environment is attributable to the vascular endothelium as opposed to the proliferative effects mediated by LOEPCs [4]. Studies have confirmed the involvement of these cells in the etiology of atherosclerosis and plaque formation. The conclusion from this research indicates that the endothelium that lines atherosclerotic plaques and the fragile neovasculature in pathological conditions related to hypertension, hypercholesterolemia, and marked pathological oxidative stress originates from endothelial and smooth muscle cells and are not derivatives of EPCs [4,15]. At the same time, patients with these high-risk oxidative states have fewer numbers of EPCs in circulation when compared to controls [16,17]. What is not clear is whether the EPCs are low secondary to oxidative stress or whether the stress is a result of low EPCs. Similarly, the vascularization of malignant tissues is related to the presence of mutated cells, and there has been no evidence of the involvement of EPCs therein [4].

Cardiovascular risk is associated with several conditions such as diabetes, hypercholesterolemia, and hypertension; in these conditions, there are several factors responsible for this increase in risk. We will discuss a few conditions and/or drugs associated with cardiovascular risk and how they influence and/or are influenced by EPCs.

Autoimmune Disorders

Chronic inflammation is associated with adverse cardiovascular incidences through the initiation and advancement of vascular dysfunction particularly in the coronary blood vessels [16-18]. Autoimmune diseases are associated with an inflammatory environment supported by the presence of elevated inflammatory markers that form the evaluative process of autoimmune diseases (such as erythrocyte sedimentation rate and C-reactive protein); the inflammatory background with elevated reactive oxygen species (ROS) fuels cardiovascular disease. ROS amplified through proinflammatory cytokines such as tumor necrosis factor-alpha (TNF-alpha) and interleukin-1-beta induce EPC apoptosis and dysfunction in a manner that is reversible in vitro by N-acetylcysteine, vitamin C, and other antioxidants.

Autoimmune diseases such as systemic sclerosis are associated with auto-antibodies to EPCs and endothelial cells. These auto-antibodies stimulate EPCs or endothelial cells in a manner that results in the production of new vessels through the release of vasculogenic factors such as interleukin-10 and VEGF by the EPCs [17]. Elevated VEGF from the EPCs and from tissues under conditions of inflammation, along with ROS work to cause a decrease in the number of EOEPCs and lead to dysfunction of LOEPCs. Endothelial stem cells transdifferentiate into mesenchymal cells in response to these factors leading to intimal thickening and luminal narrowing of coronary vessels [18] culminating into derangement of cardiac function and heart failure over a period of years.

Diabetes Mellitus

In patients with diabetes, elevated glucose levels lead to glycosylation of vascular endothelium and resultant changes in blood vessels such as narrowing and sprouting of neovasculature that is friable and at risk of rupture. Glycosylation of vascular stem cells results in the alteration of genetic processes with a resultant expansion of the adventitia, intima, and formation of abnormal blood vessels. At the same time, the EPCs that are present are dysfunctional, show a poor migratory capacity to areas in need of redress; concurrently, LOEPCs poorly differentiate and integrate into endothelial cells and newly formed vessels [19]. Pioglitazone, a drug used in the treatment of diabetes mellitus, is a peroxisome proliferator-activated receptor-gamma (PPAR-gamma) agonist which increases expression of the PPAR-gamma gene, reducing glucose and subsequently reducing CVS risk. Its effect increases the number of EPCs and reduces TNF-alpha levels. TNF-alpha increases insulin resistance by favoring a proinflammatory environment and makes control of diabetes mellitus more difficult. At the same time, TNF-alpha enhances ROS and directly induces cellular apoptosis to reduce the number and function of EPCs. Through its effect on TNF-alpha, pioglitazone enhances the efficacy of LOEPCs leading to plaque stabilization, increased migratory capacity, and enhanced adhesion of LOEPCs to new vessels, thereby reducing CVS risk. Apart from this effect, pioglitazone reduces oxidative stress through its effect on nicotinamide adenine dinucleotide phosphate (NADPH) oxidase, protecting EPCs from hydrogen peroxide-mediated cell death; this effect is extended to the rest of the tissues [20]. Enhanced oxidative stress due to ROS is responsible for the degradation of nitric oxide necessary for activation and mobilization of EPCs [19].

Hypercholesterolemia

Elevated levels of low-density lipoproteins (LDL) are linked to higher atherosclerosis and an increased risk of coronary heart disease. Oxidized LDL is responsible for inciting and perpetuating oxidative stress through a spiral of redox-based reactions via its effect of being an oxygen donor. Increased oxidative stress acts through the nitric oxide pathway to reduce the number of circulating EPCs [13], resulting in a maladaptive transformation of the coronary blood vessels and other major CVS conduits to pave the way to decompensation and development of heart failure and other CVS adverse events. The backbone of medical treatment for individuals with deranged lipid profiles is statin therapy as exemplified by pravastatin and simvastatin. Statins reduce LDL and have an anti-inflammatory effect. Pravastatin, one of the statins used for patients with high cardiovascular risk, increases mobilization and activation of LOEPCs. The enhanced effect of LOEPCs in these circumstances leads to beneficial cardiac remodeling and promotes the growth of stable endothelial cells [21].

Of note, this review revealed that levels of high-density lipoproteins (HDL) above normal do not further reduce the risk of coronary heart disease. This ties in with an effect revealed in research that elevating levels of HDL under research conditions does not increase the numbers nor the activity of EPCs beyond a physiological ceiling level. Once LDL and other lipid profiles have been normalized, there is no additional benefit in increasing HDL. An anomalous effect was found in research where higher levels of HDL led to reduced activity and function of EPCs [22].

Hypertension and Renal Failure

Hypertension is one of the most prevalent diseases in humans, and its etiology has not been fully elucidated, although some causes are well-established, such as renovascular impairment; this will be the focus of our discussion herein. Angiotensin II is formed from the cleavage of angiotensin-I primarily within the lungs; it affects cardiac myocytes along with endothelial stem cells to cause cardiac hypertrophy, endothelial damage, and proliferation of vascular smooth muscle cells. Within the kidneys, angiotensin II causes variable vasoconstriction dependent on other hormones and prostaglandins and is involved in the generation of reactive oxygen species thus maintaining a pro-inflammatory environment within the renal system of patients with renovascular disease. In these patients, there are low levels of EOEPCs and elevated levels of LOEPCs [1]. Low EOEPCs are associated with higher than normal VEGF [23,24]. Vasoconstriction sets up a low oxygen tension environment in the pathophysiologic setting of impaired renal function and is responsible for elevated levels of VEGF [23] that cause tissue hypotension, vasodilation, and abnormal remodeling of the renal vascular bed leading to increased permeability [23]. There is reason to believe angiotensin II’s pro-apoptotic nature is responsible for low levels of EOEPCs. Since LOEPCs are endothelial, and VEGF is a durability factor for endothelial cells, there is preferential survival and increase in LOEPCs in patients with renovascular and end-stage renal failure. However, these cells have impaired function and may contribute to a detrimental response to the pathophysiology related to renovascular disease [12,24]. Systemically, angiotensin-II causes an increase in ROS leading to increased senescence of EPCs. Angiotensin-converting enzyme inhibitors and angiotensin receptor blockers have been found to increase the function and numbers of EPCs when blood pressure is well-regulated to levels comparable with healthy volunteers [12] by reducing the effects of angiotensin-II.

## Conclusions

The compounding effect of obesity, cardiovascular disease, and other metabolic diseases is a threat to the function, senescence, and trans-differentiation of EPCs. These stressors are at the forefront of perpetuating damage to reparative and functional processes within the cardiovascular system and point to a need for further research into the molecular and cellular pathways relating to the function and differentiation of EPCs. The resulting new data will translate into practical therapeutic, diagnostic, and preventive approaches in the management of cardiovascular diseases and other disease states.
